# Smad4 and p53 synergize in suppressing autochthonous intestinal cancer

**DOI:** 10.1002/cam4.4533

**Published:** 2022-03-11

**Authors:** Jun Won Park, Min‐Jung Seo, Kye Soo Cho, Myeong‐Cherl Kook, Jong Min Jeong, Seul‐Gi Roh, Soo Young Cho, Jae Hee Cheon, Hark Kyun Kim

**Affiliations:** ^1^ National Cancer Center Goyang Republic of Korea; ^2^ Department of Biomedical Convergence Kangwon National University Kangwon Republic of Korea; ^3^ Department of Infectious Disease & Immunobiology Yonsei University College of Medical Science Seoul Republic of Korea; ^4^ Department of Biomedical Science and Technology Graduate School Kyung Hee University Seoul Republic of Korea

## Abstract

**Background:**

Smad4 and p53 mutations are the most common mutations in human colorectal cancers (CRCs). We evaluated whether and how they are synergistic in intestinal carcinogenesis using novel autochthonous mouse models.

**Method:**

To recapitulate human CRCs, we generated *Villin*‐*Cre*;*Smad4^F^* ^/^ *^F^*;*Trp53^F^* ^/^ *^F^* mice. We then compared the intestinal phenotype of *Villin*‐*Cre*;*Smad4^F^* ^/^ *^F^*;*Trp53^F^* ^/^ *^F^* mice (*n* = 40) with *Villin*‐*Cre*;*Smad4^F^* ^/^ *^F^* (*n* = 30) and *Villin*‐*Cre*;*Trp53^F^* ^/^ *^F^* mice (*n* = 45).

**Results:**

Twenty‐week‐old *Villin*‐*Cre*;*Smad4^F^* ^/^ *^F^*;*Trp53^F^* ^/^ *^F^* mice displayed spontaneous highly proliferative intestinal tumors, and 85% of mice developed adenocarcinomas. p21 was downregulated in the intestinal mucosa in *Villin*‐*Cre*;*Smad4^F^* ^/^ *^F^*;*Trp53^F^* ^/^ *^F^* mice than in *Villin*‐*Cre*;*Smad4^F^* ^/^ *^F^* and *Villin*‐*Cre*;*Trp53^F^* ^/^ *^F^* mice. *Villin*‐*Cre*;*Smad4^F^* ^/^ *^F^*;*Trp53^F^* ^/^ *^F^* mice displayed multistep intestinal tumorigenesis and Wnt activation. Long‐term CWP232291 (small‐molecule Wnt inhibitor) treatment of *Villin*‐*Cre*;*Smad4^F^* ^/^ *^F^*;*Trp53^F^* ^/^ *^F^* mice suppressed intestinal tumorigenesis and progression. CWP232291 treatment downregulated cancer stem cell (CSC) tumor markers including CD133, Lgr‐5, and Sca‐1. CWP232291 treatment reduced the CSC frequency. Small‐molecule Wnt inhibitors reduced intestinal CSC populations and inhibited their growth, along with Bcl‐X_L_ downregulation. Furthermore, BH3I‐1, a Bcl‐X_L_ antagonist, increasingly inhibited intestinal CSCs than bulk tumor cells.

**Conclusion:**

Smad4 loss and p53 loss are synergistic in autochthonous intestinal carcinogenesis, by downregulating p21 and activating Wnt/β‐catenin pathway.

## Graphical abstract

Cooperation of Smad4 and p53 in constraining the intestinal tumor development and progression. Loss of Trp53 and Smad4 is synergistic in spontaneous mouse intestinal carcinogenesis and unrecognized therapeutic vulnerabilities. Wnt inhibitors and TGF‐β inhibitors provide therapeutic benefit to mice‐bearing colorectal tumors, as monotherapy or in combination with immune checkpoint inhibitors.

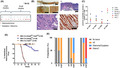

## 1 INTRODUCTION

Colorectal cancer (CRC) is the third most common cancer worldwide.^1^ Whereas *SMAD4* and *TP53* inactivating mutations are very frequent in human colon cancer, with mutation rates of 14% and 64%, respectively (www.cbioportal.org), it has not been fully elucidated whether and how they interact in colorectal carcinogenesis. Therefore, we performed intestinal epithelium‐specific knockout of Smad4 and p53, to evaluate the molecular mechanism of synergy and potential therapeutic vulnerabilities created by loss of these two genes.

Tumor suppressor gene *SMAD4* is frequently mutated or silenced during tumor initiation and development.^2^ Also, loss of *SMAD4* expression is reported in many solid tumors including CRC, leading to enhanced cancer cell proliferation.^3^, ^4^ Smad4 is a key signal transducer in the transforming growth factor beta (TGF‐β) and bone morphogenetic protein (BMP) signaling pathways, and its downregulation is associated with a decreased overall and disease‐free survival.^5^ Smad4 deficiency promotes intestinal tumorigenesis and metastasis in mice.^6^, ^7^, ^8^, ^9^, ^10^ Smad4 haploinsufficiency reportedly affects mouse intestinal tumorigenesis and progression and Smad4 deletion in combination with genetic alterations in antigen‐presenting cell (APC) results in intestinal cancer in mice.^9^, ^10^ Smad4 deletion along with an *APC* alteration results in intestinal cancer in mice.^9^ Smad4‐mediated BMP signaling inhibits intestinal tumorigenesis,^6^ while Smad4‐independent BMP signaling promotes metastasis in colorectal tumors.^8^ p53 is an important tumor suppressor that maintains genome stability and integrity, inhibits the cell cycle, and induces apoptosis.^11^ p53 encodes a protein that regulates the cell cycle, DNA repair, senescence, and apoptosis.^12^ p53‐null mice are at an increased risk of *Apc* mutation‐induced intestinal tumorigenesis,^13^ whereas no study has evaluated in vivo phenotype resulting from intestinal epithelium‐specific knockout of both p53 and Smad4.

p21, cyclin‐dependent kinases inhibitor 1A (CDKN1A), is one of the most important downstream mediators of p53. P21 is a negative cell cycle regulator and induces senescence.^14^ Loss of p21 is a poor prognostic factor in CRC.^15^

Using our expertise in genetically engineered mouse models,^16^, ^17^ here we demonstrate that Wnt/β‐catenin pathway mediates the autochthonous intestinal carcinogenesis in mice deficient in Smad4 and p53. β‐catenin activation leads to the initiation, progression, metastasis, drug resistance, and evasion of apoptosis of cancer cells.^18^ β‐catenin coactivates T‐cell factor (TCF)/lymphocyte enhancer factor, leading to the upregulation of oncogenic Wnt‐related target genes.^18^ Alteration of the Wnt/β‐catenin pathway is associated with initiation, progression, metastasis, and maintenance of cancer stem cells (CSCs) in CRC.^19^ We suggest that Wnt/β‐catenin signaling inhibition can be a potential chemo‐preventive strategy for human CRCs deficient in Smad4 and p53.

## 2 MATERIALS AND METHODS

### 2.1 Mice

Mouse studies were performed under the approval of the Animal Care and Use Committees of Korea National Cancer Center. *Villin*‐*Cre* (B6.Cg‐Tg(Vil‐Cre)20Sy) and *Trp53^F^* ^/^ *^F^* (FVB.129‐Trp53tm1Brn) mice were provided by the Mouse Models of Human Cancers Consortium at the NCI Frederick Cancer Research Center. *Smad4^F^* ^/^ *^F^* mice were provided by Dr. Chuxia Deng.^20^ We monitored *Villin*‐*Cre*‐positive mice until they became moribund or showed stress signs, when necropsies were performed. Carcinoma‐free intervals were compared by the log‐rank test using GraphPad Prism 5 (GraphPad Software, http://www.graphpad.com). CWP232291 was provided by JW Pharmaceutical.

### 2.2 Immunostaining and immunoblot analysis

We performed immunohistochemistry (IHC) analyses on primary mouse tissues using ImmPRESS Peroxidase Polymer kit (Vector Laboratories) according to the method of our previous report.^21^ The following primary antibodies were used in this study; p53 (sc‐6243; Santa Cruz), β‐catenin (610154; BD Biosciences), Smad4 (sc‐7966; Santa Cruz), Ki‐67 (ab16667; Abcam), p21 (sc‐398; Santa Cruz), proliferating cell nuclear antigen (PCNA, sc‐56; Santa Cruz), p27 (sc‐528; Santa Cruz), p15 (#4822; Cell Signaling), Myc (ab32072; Abcam), and cyclin D1 (#2978; Cell Signaling). To perform immunofluorescence (IF) on mouse primary tissues, frozen tissues sections were fixed with 4% paraformaldehyde, blocked with phosphate‐buffered saline (PBS) containing 5% normal goat serum, and then incubated with Sca‐1 (108101; BioLegend), CD133 (12‐1331‐82; eBioScience), and Lgr‐5 (ab75732; Abcam) overnight at 4°C. IgG Texas red (TI‐9400; Vector Laboratories) and IgG Alexa Fluor 488 (A‐11008; Thermo Fisher Scientific) were used for secondary antibodies. Slides were mounted with VECTASHIELD mounting media (H‐1200; Vector Laboratories).

IHC grading was performed under high‐power microscopic magnification (×400). The positive rates were depicted as the mean value of at least five high‐power fields. Myc, cyclin D1, and nuclear β‐catenin expression were scored according to the percentage of cancer cells exhibiting unequivocal moderate to strong nuclear staining. The IF scoring for CD133, Sca‐1, and Lgr‐5 expression was based on the percentage of cancer cells showing the membranous immunoreactivity.

For bromodeoxyuridine (BrdU) assays, mice were intraperitoneally injected with BrdU (Sigma) at 20 mg/kg body weight. Two hours later, the mice were euthanized, and the intestine was dissected and fixed in 10% PBS‐buffered formalin before embedding in paraffin. The BrdU immunostaining was carried out using a BrdU Detection IHC kit (2760; Chemicon).

For western blot analysis, total cell and tissue extracts were fractionated by electrophoresis on a gradient sodium dodecyl sulfate–polyacrylamide gel and transferred onto a polyvinylidene fluoride membrane according to the method of our previous report.^21^ The following primary antibodies were used; p53 (sc‐6243; Santa Cruz), Smad4 (sc‐7966; Santa Cruz), Bcl‐X_L_ (sc‐8392; Santa Cruz), Bcl‐2 (sc‐492; Santa Cruz), and GAPDH (sc‐32233; Santa Cruz). Immunodetection was performed using an enhanced chemiluminescence detection kit (Thermo Fisher Scientific).

### 2.3 TUNEL assay

Apoptotic cells in tumor tissues were measured using the terminal deoxynucleotidyl transferase dUTP nick end labeling (TUNEL) assay employing the Fluorescein FragEL^TM^ DNA Fragmentation Detection Kit (QIA39; Calbiochem) according to the manufacturer's instructions. Stained slides were evaluated using Zeiss Axio Imager HBO 100 (Carl Zeiss).

### 2.4 Flow cytometry

Single cell suspension dissociated from S1M allografts and primary mouse cells were stained with Sca‐1 FITC (553335; BD Pharmingen), CD133 PE (12‐1331‐82; eBioScience), CD44 FITC (11‐0441‐81; eBioScience), CD45 PE‐CyTM7 (552848; BD Pharmingen), rat IgG isotype control PE (553930; BD Pharmingen), and rat IgG isotype control FITC (11‐4031‐81; eBioScience) for 1 h at 4°C in the dark room. The cells were analyzed on FACS Calibur (BD Biosciences) and sorted on an Aria cell sorter (BD Biosciences).

### 2.5 DNA microarray and quantitative real‐time RT‐PCR analyses

DNA microarray was conducted using total RNA isolated from frozen allograft tissue, as recommended by the manufacturer (Mouse Genome 430A 2.0; Affymetrix). For consensus molecular subgroups (CMS) typing, “randomForest” package was used (RStudio, version 1.2.1335).

RT‐PCR reactions were performed on a Roche LC480 (Roche Diagnostics) using QuantiTect SYBR Green PCR Master Mix (Qiagen).

PCR primers were F: 5′‐TTG CAC TCT GGT GTC TGA G‐3′; R: 5′‐AAT CTG TCA GGC TGG TCT G‐3′ for *Cdkn1a*, F: 5′‐CGATAGAGGAGCATAGAAAGCAC‐3′; R: 5′‐GCTCTCTGTCTGTCCAGTTTC‐3′ for *Birc5*, and F: 5′‐GGTCGGTGTGAACGGATTTG‐3′; R: 5′‐GTGAGTGGAGTCATACTGGAAC‐3′ for *Gapdh*.

### 2.6 Statistics

Statistical analysis was performed by GraphPad Prism 5 (GraphPad Software, http://www.graphpad.com). An analysis was performed using a Student's *t*‐test. Carcinoma‐free intervals and the survival differences between cohorts were assessed by the log‐rank test. *p* values of less than 0.05 were considered statistically significant.

## 3 RESULTS

### 3.1 Smad4 and p53 are synergistic in suppressing the development and progression of autochthonous intestinal adenocarcinoma

We compared the intestinal phenotype of *Villin*‐*Cre*; *Smad4^F^* ^/^ *^F^*; *Trp53^F^* ^/^ *^F^* mice (*n* = 40) with *Villin*‐*Cre*; *Smad4^F^* ^/^ *^F^* (*n* = 30) and *Villin*‐*Cre*; *Trp53^F^* ^/^ *^F^* mice (*n* = 45). As expected, no Smad4 and p53 immunoreactivity was observed in the normal intestinal epithelium and tumors arising in each genotype (Figure S1). *Villin‐Cre;Smad4* *^F/F^* *;Trp53* *^F/F^* mice tumor shows the characteristics of adenoma/dysplasia, acinar adenocarcinoma and mucinous adenocarcinoma in hematoxylin and eosin (H&E) staining (Figure S2).

**FIGURE 1:**
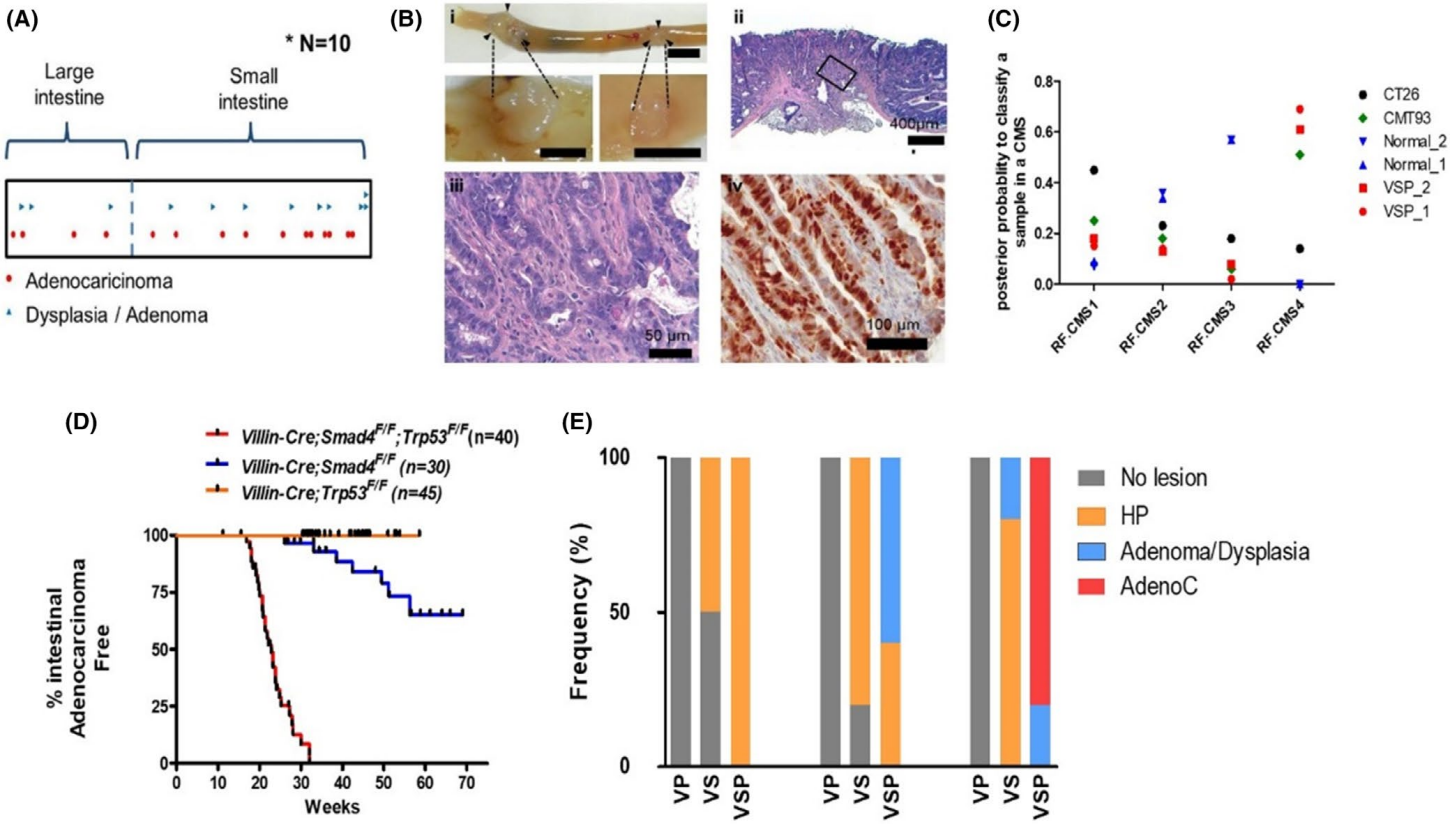
Cooperation of Smad4 and p53 in constraining the intestinal tumor development and progression. *V*;*P*; *Villin*‐*Cre*; *Trp53* ^F/F^, *V*;*S*; *Villin*‐*Cre*; *Smad4^F^* ^/^ *^F^*, *V*;*S*;*P*; *Villin*‐*Cre*; *Smad4^F^* ^/^ *^F^*; *Trp53^F^* ^/^ *^F^*. (A) Distribution and localization of intestinal tumors on intestinal track from 20‐week‐old *V*;*S*;*P* mice. All tumors arising from 10 *V*;*S*;*P* mice are accumulatively shown. (B) Representative images for gross and microscopic findings of *V*;*S*;*P* intestinal cancers. (B[i]) Gross findings. Bar = 1 cm. Arrow heads indicate tumor margins. (B[ii, iii]) Histological findings based on H&E slides. A boxed area in (B[ii]) was magnified in (B[ii]). (B[iv]) A representative image of IHC staining for Ki‐67 in a cancer tissue. (C) Determination of the CMS type of tumors formed in *Villin*‐*cre*; *Smad4^F^* ^/^ *^F^*; *Trp53^F^* ^/^ *^F^* mice. (D) Intestinal adenocarcinoma‐free survival of each genotype. *V*;*S*;*P*, *n* = 40; *V*;*S*, *n* = 30; *V*;*P*, *n* = 45. (E) Incidence of pre‐neoplastic and neoplastic lesions from each genotype mice (*n* = 5) at 10, 15, and 20 weeks of age. AdenoC, adenocarcinoma; CMS, consensus molecular subgroups; H&E, hematoxylin and eosin; HP, hyperplastic polyp; IHC, immunohistochemistry

The median survival duration of *Villin*‐*Cre*; *Smad4^F^* ^/^ *^F^*; *Trp53^F^* ^/^ *^F^* mice was 21.4 week. The most common cause of death among these mice was a duodenal obstruction, followed by colic/enteric intussusception. Adenocarcinoma was not detected in other organs including the stomach, lungs, and spleen in the *Villin*‐*Cre*; *Smad4^F^* ^/^ *^F^*; *Trp53^F^* ^/^ *^F^*, *Villin*‐*Cre*; *Smad4^F^* ^/^ *^F^*, and *Villin*‐*Cre*; *Trp53^F^* ^/^ *^F^* mice. Median number of tumors was 3.7 per *Villin*‐*Cre*; *Smad4^F^* ^/^ *^F^*; *Trp53^F^* ^/^ *^F^* mouse at 20 weeks of age, with a mean size of 3.9 mm and widespread intestinal distribution (Figure 1A). Overall, intestinal cancers protruded into the lumen and/or presented transmural invasion (Figure 1B[i]). Histological analysis revealed that tumors were moderately differentiated adenocarcinoma displaying desmoplastic responses, often with necrotic debris in the gland and infiltrating niches of tumor cells (Figure 1B[ii]). The cancer cells invaded the muscle layer (Figure 1B[iii]). IHC for Ki‐67 revealed highly proliferative cancer cells (Figure 1B[iv]). Tumors formed in *Villin*‐*Cre*; *Smad4^F^* ^/^ *^F^*; *Trp53^F^* ^/^ *^F^* mice were classified as the CMS type 4, according to DNA microarray analysis (Figure 1C).

Twenty‐nine of 35 *Villin*‐*Cre*; *Smad4^F^* ^/^ *^F^*; *Trp53^F^* ^/^ *^F^* mice (82.9%) developed multiple spontaneous intestinal adenocarcinomas until death (median adenocarcinoma‐free survival, 5.96 months), while 7 of 30 *Villin*‐*Cre*; *Smad4^F^* ^/^ *^F^* mice (23.3%) developed adenocarcinomas (median adenocarcinoma‐free survival, 14.07 months) (Figure 1D). Although all *Villin*‐*Cre*; *Trp53^F^* ^/^ *^F^* mice died of systemic lymphoma (median survival, 9.9 months) without evidence of epithelial tumors (Figure 1D), histopathological examination revealed pre‐malignant lesions in the intestinal mucosa at 10, 15, and 20 weeks of age (Figure 1E). *Villin*‐*Cre*; *Smad4^F^* ^/^ *^F^* mice exhibited delayed tumorigenesis and progression compared with *Villin*‐*Cre*; *Smad4^F^* ^/^ *^F^*; *Trp53^F^* ^/^ *^F^* mice, implying that p53 suppresses intestinal tumorigenesis and progression in a Smad4 null background (Figure 1E).

### 3.2 p21 mediates the synergistic tumor suppression of Smad4 and p53

**FIGURE 2:**
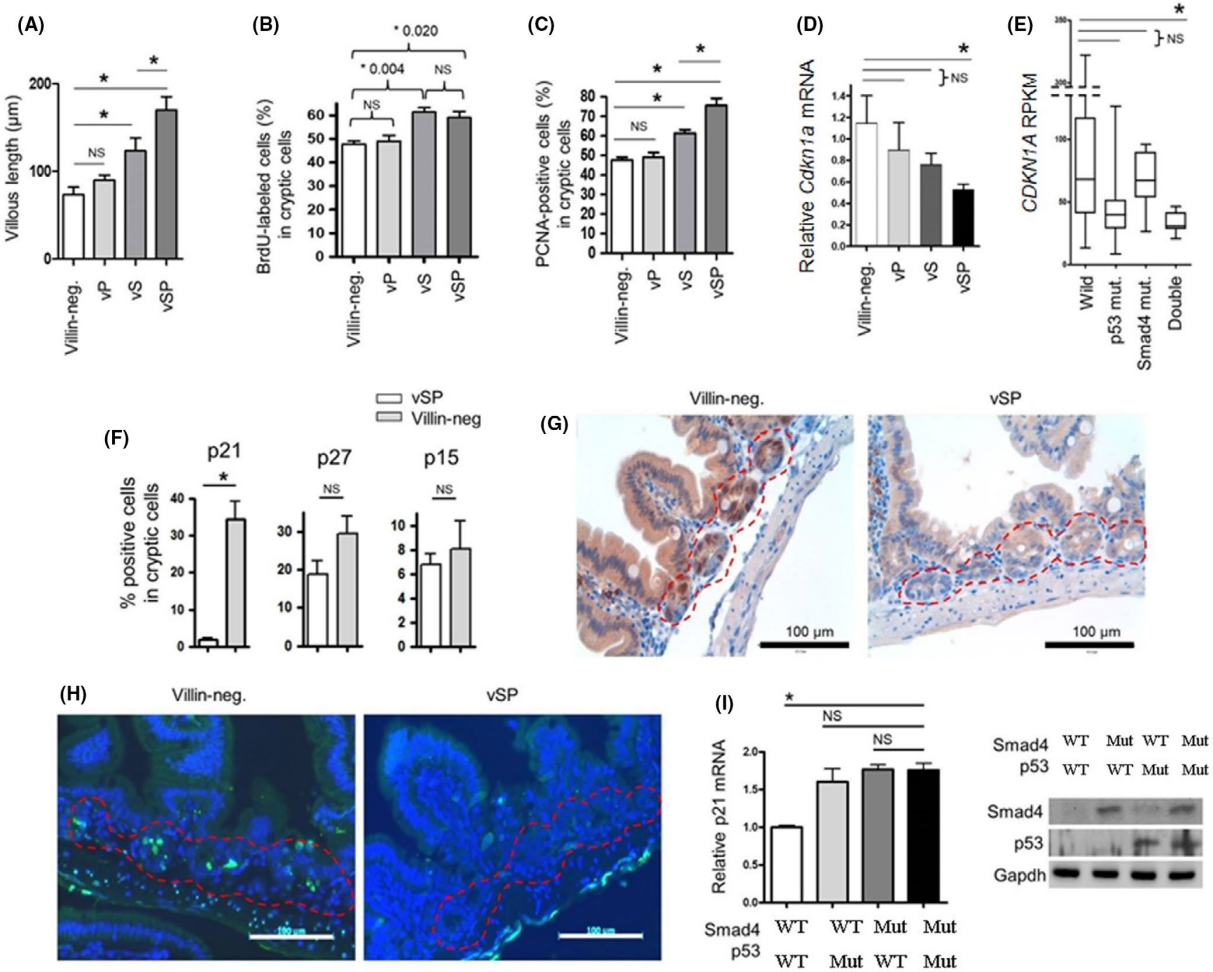
Cooperation of Smad4 and p53 in regulating normal intestinal mucosal growth. *VSP*, *Villin*‐*Cre*; *Smad4^F^* ^/^ *^F^*; *Trp53^F^* ^/^ *^F^*. *VS*, *Villin*‐*Cre*; *Smad4^F^* ^/^ *^F^*. *VP*, *Villin*‐*Cre*; *Trp53^F^* ^/^ *^F^*. (A) Villous length of normal intestinal mucosa from each genotype mice (*n* = 5) at 20 weeks of age based on H&E staining slides. (B) BrdU staining results in BrdU‐labeled cryptic cells of intestinal mucosa from each genotype (*n* = 5). Mice were intraperitoneally injected with BrdU at 20 mg/kg body weight. Two hours later, the intestinal mucosa was collected. (C) The percentage of PCNA‐positive cells in cryptic cells of normal intestinal mucosa from each genotype mice (*n* = 5) at 20 weeks of age. (D) p21 mRNA expression in mouse normal intestinal epithelium from each genotype mice (*n* = 5) at 20 weeks of age based on quantitative real‐time PCR analysis. (E) mRNA expression of p21 in human colorectal cancer TCGA dataset, according to Smad4 and p53 status. (F) The percentages of p21‐, p27‐, and p15‐positive cells in cryptic cells from 10‐week‐old *Villin*‐*Cre*; *Smad4^F^* ^/^ *^F^*; *Trp53^F^* ^/^ *^F^* (*n* = 5) and *Villin*‐*Cre*‐negative control mice (*n* = 5) after 24 h of 10 Gy of IR treatment based on IHC staining results. (G) Representative IHC images for p21 expression in IR‐treated intestinal mucosa from *Villin*‐*Cre*; *Smad4^F^* ^/^ *^F^*; *Trp53^F^* ^/^ *^F^* and *Villin*‐*Cre*‐negative control mice. Red dotted lines indicate crypt cells. (H) Representative images for TUNEL assay in IR‐treated intestinal mucosa from of *Villin*‐*Cre*; *Smad4^F^* ^/^ *^F^*; *Trp53^F^* ^/^ *^F^* and *Villin*‐*Cre*‐negative control mice. Nucleus, blue (DAPI); Apoptotic cells, green. (I) Quantitative real‐time PCR analysis for the measurement of etoposide‐induced p21 mRNA expression in primarily cultured *Villin*‐*Cre*; *Smad4^F^* ^/^ *^F^*; *Trp53^F^* ^/^ *^F^* intestinal cancer cells (primary #1) after the restoration of Smad4 and/or p53 using lentiviral system. (Right) Western blotting analysis. BrdU, bromodeoxyuridine; H&E, hematoxylin and eosin; IHC, immunohistochemistry; IR, ionizing radiation; NS, No significance; PCNA, proliferating cell nuclear antigen. *p < 0.05

We then addressed the molecular mechanism of synergy in tumor suppression between Smad4 and p53. The PCNA positivity rate was higher in cryptic cells of *Villin*‐*Cre*; *Smad4^F^* ^/^ *^F^*; *Trp53^F^* ^/^ *^F^* mice than in those of *Villin*‐*Cre*; *Smad4^F^* ^/^ *^F^* mice (Figure 2A–C). Both Smad4 and p53 serve as transcription factors regulating p21 (*Cdkn1a*),^11^ which in turn regulates cell cycle arrest and apoptosis through its interaction with PCNA, an essential cofactor for DNA polymerases.^22^ Indeed, *Cdkn1a* mRNA was significantly downregulated in histologically normal small intestinal mucosa in *Villin*‐*Cre*; *Smad4^F^* ^/^ *^F^*; *Trp53^F^* ^/^ *^F^* mice rather than in mice harboring other genotypes at 10‐week postpartum (Figure 2D). According to TCGA dataset of human CRC,^23^ *CDKN1A* mRNA was significantly downregulated in tumors harboring both p53 and Smad4 mutations rather than in those with intact Smad4 and p53 (Figure 2E).

To investigate the reciprocal roles of Smad4 and p53 in p21 induction under apoptotic conditions, we treated *Villin*‐*Cre*; *Smad4^F^* ^/^ *^F^*; *Trp53^F^* ^/^ *^F^* and *Villin*‐*Cre*‐*negative* mice with ionizing radiation (IR) of 10 Gy. After 24 h of IR, cryptic cells in *Villin*‐*Cre*; *Smad4^F^* ^/^ *^F^*; *Trp53^F^* ^/^ *^F^* mice presented significantly blunted p21 induction compared to those in *Villin*‐*Cre*‐*negative* mice according to IHC (Figure 2F,G). The TUNEL assay revealed a reduction in apoptosis among the cryptic cells of *Villin*‐*Cre*; *Smad4^F^* ^/^ *^F^*; *Trp53^F^* ^/^ *^F^* mice in comparison with *Villin*‐*cre*‐*negative* mice (Figure 2H). Furthermore, primary cultures of *Villin*‐*Cre*; *Smad4^F^* ^/^ *^F^*; *Trp53^F^* ^/^ *^F^* intestinal cancer cells (primary #1) revealed a reduction in p21 induction after etoposide treatment in comparison with Smad4‐ and/or p53‐restored cells (Figure 2I), implying the reciprocal regulation of p21 by Smad4 and p53. Together, these results show that Smad4 and p53 suppress tumorigenesis and progression partially through p21‐induced apoptosis.

### 3.3 Smad4 and p53 suppress intestinal carcinogenesis by inactivating Wnt/β‐catenin signaling

**FIGURE 3:**
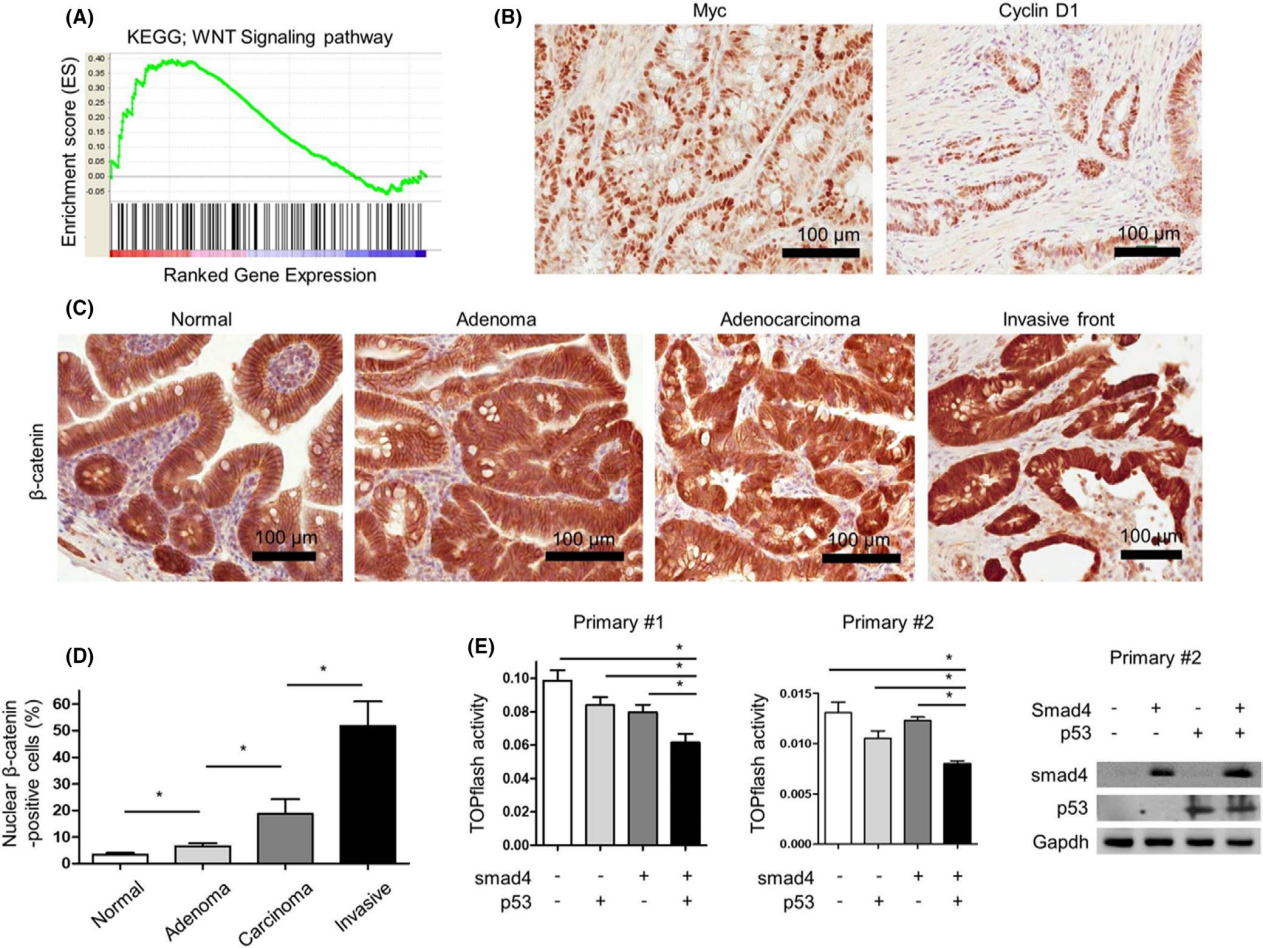
The activation of Wnt signaling pathway in tumors arising from *Villin*‐*Cre*; *Smad4^F^* ^/^ *^F^*; *Trp53^F^* ^/^ *^F^* mice. (A) The enrichment of Wnt signaling pathway using GSEA in *Villin*‐*Cre*; *Smad4^F^* ^/^ *^F^*; *Trp53^F^* ^/^ *^F^* intestinal adenocarcinomas (*n* = 2) compared with Villin‐Cre‐negative normal intestinal mucosa (*n* = 2) based on mouse gene expression microarray analysis. (B) Representative IHC images for c‐myc and cyclin D1 in primary *Villin*‐*Cre*; *Smad4^F^* ^/^ *^F^*; *Trp53^F^* ^/^ *^F^* intestinal cancer tissues. (C) Representative IHC images for β‐catenin in neoplastic lesions from *Villin*‐*Cre*; *Smad4^F^* ^/^ *^F^*; *Trp53^F^* ^/^ *^F^* mice. (D) IHC grading of nuclear β‐catenin expression in neoplastic lesions from *Villin*‐*Cre*; *Smad4^F^* ^/^ *^F^*; *Trp53^F^* ^/^ *^F^* mice (*n* = 3). (E) TOPflash reporter activities in primarily cultured cancer cell lines (primary #1 and primary #2) established from *Villin*‐*Cre*; *Smad4^F^* ^/^ *^F^*; *Trp53^F^* ^/^ *^F^* intestinal adenocarcinomas to measure Wnt pathway activities after the restoration of Smad4 and/or p53 using lentiviral system. Right, western blot analysis to confirm the restoration of Smad4 and/or p53 in primary #2 cells. GSEA, gene set enrichment analysis; IHC, immunohistochemistry. *p < 0.05

To identify the signaling pathways associated with the spontaneous intestinal tumorigenesis, we compared DNA microarray data between the adenocarcinomas (*n* = 2) formed in *Villin*‐*Cre*; *Smad4^F^* ^/^ *^F^*; *Trp53^F^* ^/^ *^F^* mice and *Villin*‐*Cre*‐negative normal intestinal mucosae (*n* = 2). Gene set enrichment analysis (GSEA) revealed that the Wnt signaling pathway was significantly enriched (Figure 3A; Table S1). Genes upregulated by >1.5‐fold in cancer tissues rather than in normal intestinal mucosae include Wnt signaling‐related genes such as *Fzd1* (fold change [FC], 5.6), *Myc* (FC, 2.6), *Ccnd1* (FC, 1.6), *Mmp2* (FC, 10.4), *Mmp7* (FC, 16.0), *Mmp8* (FC, 12.9), *Mmp12* (FC, 52.2), and *Spp1* (FC, 149.3). IHC revealed the upregulation of Myc, cyclin D1, and Wnt target genes in cancer cells (Figure 3B). Nuclear β‐catenin accumulation increased as intestinal lesions progressed from benign to malignant (Figure 3C,D).

Smad4 and p53 rescue in primary intestinal cancer cell lines (primary #1 and primary #2) significantly reduced Wnt/β‐catenin signaling reporter activity in the primary cancer cells, and the inhibitory effects were greater than those of individual restoration of Smad4 or p53 (Figure 3E).

### 3.4 Pharmacologic inhibition of Wnt/β‐catenin signaling suppressed the development and progression of autochthonous intestinal cancer in the background of Smad4 loss and p53 loss

Treatment with various Wnt/β‐catenin inhibitors, such as CCT031374 and β‐catenin/Tcf inhibitors II and V, suppressed in vitro proliferation and β‐catenin reporter activity of *Villin*‐*Cre*; *Smad4^F^* ^/^ *^F^*; *Trp53^F^* ^/^ *^F^* cells primary cultured from autochthonous intestinal cancers (primary #1 cells) (Figure S3A). Human CRC cell lines SW620 and COLO205 also demonstrated reduced in vitro proliferation and cyclin D1 protein expression after exposure to CWP232291, a Wnt/β‐catenin inhibitor (JW Pharmaceutical; U.S. patent 8,940,739) (Figure S3A,B).^24^ After 24 h intraperitoneal administration with 100 mg/kg of CWP232291, survivin (*Birc5*), a Wnt target gene, was downregulated in normal intestinal mucosa (Figure S3D).

**FIGURE 4:**
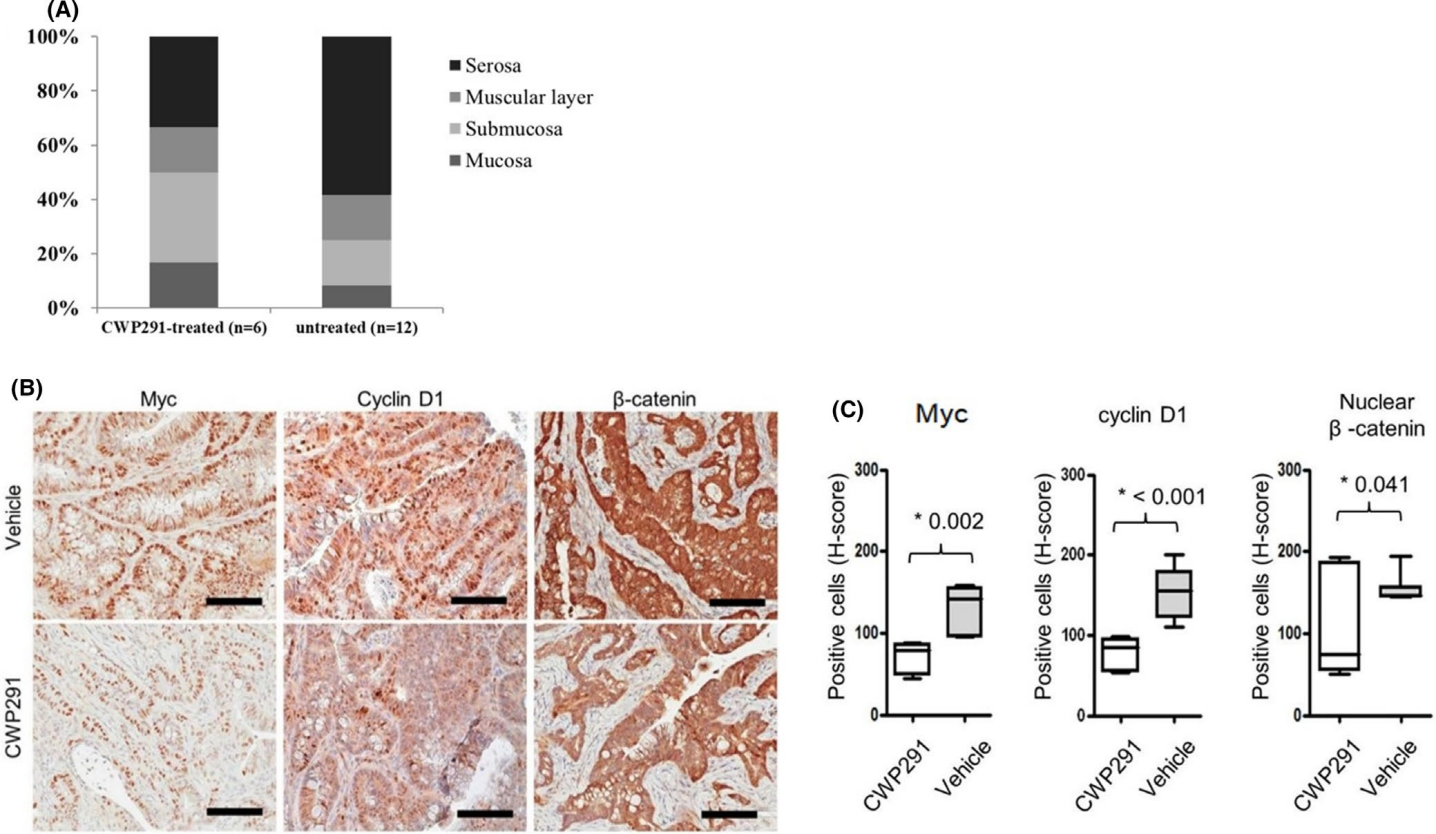
(A) Proportion of primary intestinal tumors invasive into submucosa, muscular layer, and serosa after 100 mg/kg CWP232291 treatment of *Villin*‐*Cre*; *Smad4^F^* ^/^ *^F^*; *Trp53^F^* ^/^ *^F^* mice twice per week for 17 weeks. (B, C) In vivo limiting dilution assay in SCID mice after CWP232291 treatment. TIC frequency was calculated and *p* value was measured using ELDA web tool. TIC, tumor‐initiating cell

We then evaluated whether long‐term in vivo treatment with CWP232291 could suppress the development of autochthonous intestinal cancer in *Villin*‐*Cre*; *Smad4^F^* ^/^ *^F^*; *Trp53^F^* ^/^ *^F^* mice. To this aim, 3‐week‐old *Villin*‐*Cre*; *Smad4^F^* ^/^ *^F^*; *Trp53^F^* ^/^ *^F^* mice were intraperitoneally injected biweekly with either 100 mg/kg of CWP232291 (*n* = 19) or normal saline (*n* = 27) for 17 weeks (Table 1). Histological assessment revealed lower numbers of tumor‐bearing mice in CWP232291 treatment group than in no treatment group (50.0% vs. 84.0% with vehicle only; *p* < 0.01). More importantly, CWP232291 significantly decreased the incidence (37.5% vs. 78.3% with vehicle only; *p* < 0.05) and invasiveness (Figure 4A) of malignant tumors. Tumor multiplicity was also lower in CWP232291‐treated mice (1.2 ± 0.4 vs. 2.8 ± 0.4 with vehicle only; *p* < 0.01). CWP232291 treatment reduced β‐catenin, MYC, and cyclin D1 immunostaining in tumors (Figure 4B,C). These results indicate that CWP232291 inhibits Wnt signaling by downregulating β‐catenin and Wnt target genes, resulting in the suppression of intestinal tumorigenesis and progression.

**TABLE 1:** Incidence and multiplicity of autochthonous intestinal tumor arising from *Villin*‐*cre*; *Smad4^F^* ^/^ *^F^*; *Trp53^F^* ^/^ *^F^* mice after CWP232291 treatment

Group	CWP232291 treated	Untreated
Total mice (*n*)	19	27
Dead mice before 20 weeks (*n*)	3 (adenoma, *n* = 2; unknown, *n* = 1)	4 (adenoma, *n* = 1; hyperplasia, *n* = 1; unknown, *n* = 2)
Remaining mice until 20 weeks (*n*)	16	23
Tumor‐bearing mice [*n* (%)]	9 (50.0)**	21 (84.0)
AdenoC. mice [*n* (%)]	6 (37.5)*	18 (78.3)
Tumor multiplicity	1.18 ± 0.36**	2.83 ± 0.39
Tumor diameter (mm)	4.25 ± 0.52	5.53 ± 0.97

* *p* < 0.05.

** *p* < 0.01.

### 3.5 Smad4 and p53 suppress intestinal carcinogenesis by decreasing CSC population

**FIGURE 5:**
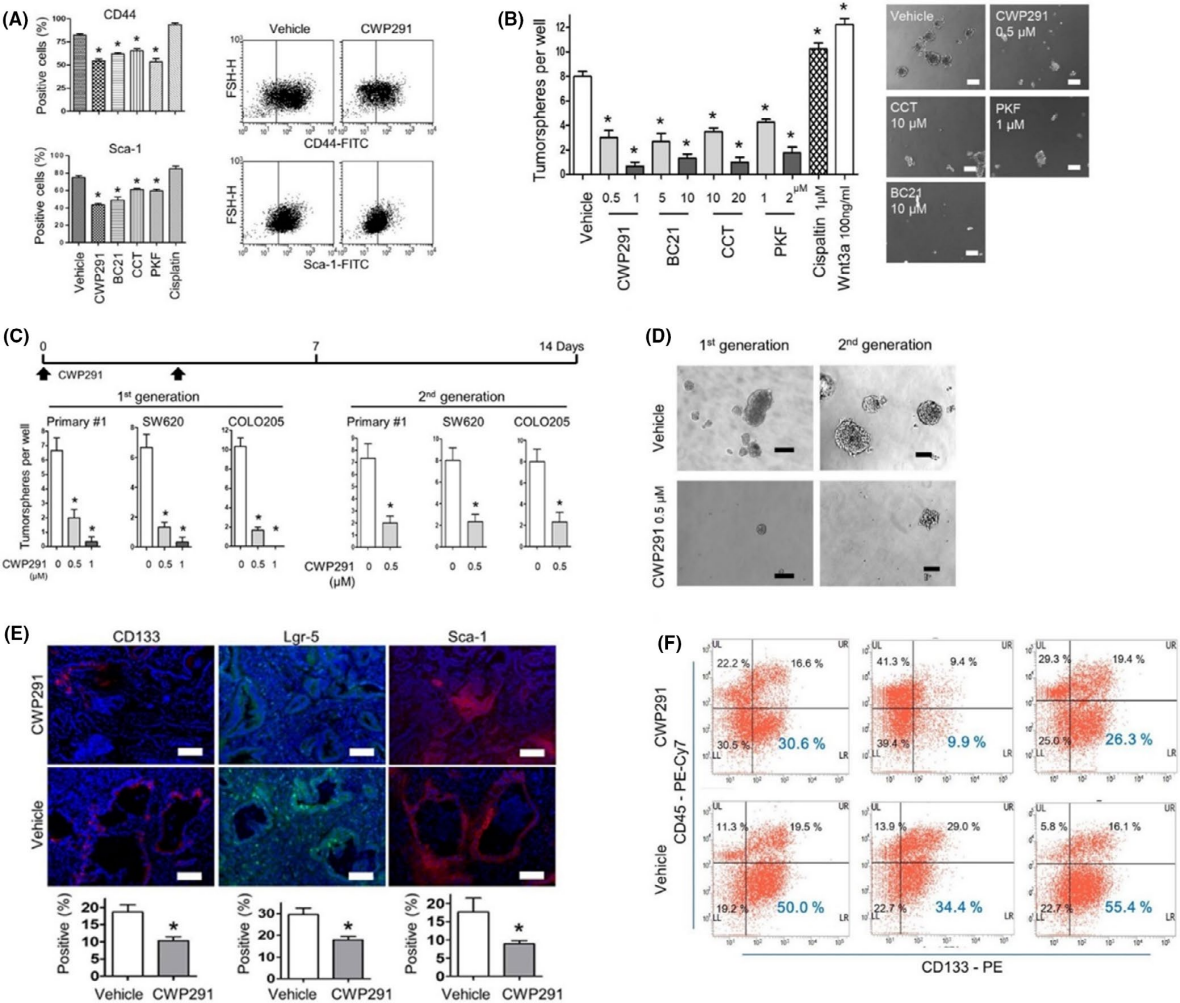
(A) FACS analysis for CD44 or Sca‐1‐positive subpopulations in primary #1 cells after Wnt small‐molecule inhibitors for 24 h. CWP291, 1 µM CWP232291; BC21, 10 µM BC21; CCT, 20 µM CCT031374; PKF, 2 µM PKF 118–774; Cisplatin, 1 µM cisplatin. Right, representative FACS images showing reduced CD44 or Sca‐1‐positive cells in primary #1 cells after CWP232291 treatment. (B) Sphere‐forming assay showing reduced tumorsphere formation from primary #1 cells after the treatment for 7 days with Wnt inhibitors. The spheres greater than 100 mm of diameter were enumerated. Right, representative images of tumor spheres after the treatment with Wnt inhibitors. (C) CWP232291 inhibited primary (with CWP232291) and second sphere formation (without CWP232291) in primary #1 and SW620. (D) Representative images of tumorspheres from primary #1 cells of (C). (E) Representative IF images for CD133, Lgr‐5, and Sca‐1, putative mouse intestinal cancer stem cell markers, in vehicle‐ or CWP232291‐treated allografts of SCID mice. SCID mice injected with primary #1 cell were treated with 100 mg/kg CWP232291 twice a week for 8 weeks. Bottom, IF scoring for CD133, Lgr‐5, and Sca‐1‐positive tumor cells in vehicle‐ (*n* = 3) or CWP232291‐treated (*n* = 3) allografts. Bar = 100 µm. (F) FACS analysis for CD133 in CWP232291‐ or vehicle‐treated allografts. Single cell suspensions were obtained from allografts after dispase digestion. FACS, fluorescence‐activated cell sorting; IF, immunofluorescence. *p < 0.05

Given that Wnt/β‐catenin signaling maintains CSCs,^25^, ^26^ we evaluated whether pharmacologic inhibition of Wnt/β‐catenin signaling reduces CSCs in intestinal tumors deficient in Smad4 and p53. Treatment of primary #1 cells with a series of Wnt inhibitors reduced the CD44‐ or Sca‐1‐positive subpopulation and tumorsphere formation (Figure 5A,B). CWP232291 treatment disrupted secondary tumorsphere formation from primary tumorspheres of primary #1 cells and human CRC cell lines SW620 and COLO205 (Figure 5C,D).

We then conduced in vivo limiting dilution assays for tumor‐initiating cells (TICs). The frequency of TICs was significantly lower in CWP232291 (100 mg/kg for 8 weeks)‐treated group (1/48,069) than in untreated group (1/3223) (*p* < 0.001). IFs of CD133 and Lgr‐5, which are colon CSC markers, were weaker in CWP232291‐treated allografts than in untreated allografts (Figure 5E). Fluorescence‐activated cell sorting analysis showed decrease in CD133‐positive subpopulation in CWP232291‐treated allografts than in untreated allografts (Figure 5F).

We previously reported that Sca‐1 is a mouse gastric CSC marker.^27^ According to in vivo limiting dilution assay, Sca‐1^high^ population of *Villin*‐*Cre*; *Smad4^F^* ^/^ *^F^*; *Trp53^F^* ^/^ *^F^* primary cultured cells demonstrated higher tumorigenic potential than Sca‐1^negative/low^ cells, suggesting that Sca‐1 may be a mouse colorectal CSC marker (Table S2). Also, Table S3 shows pathways which downregulated by CWP233291‐treated allograft compared with vehicle‐treated allograft (Table S3). Notably, in vivo CWP232291 treatment reduced the Sca‐1‐positive population in *Villin*‐*Cre*; *Smad4^F^* ^/^ *^F^*; *Trp53^F^* ^/^ *^F^* allograft (Figure 5F). These results collectively suggest that Wnt/β‐catenin‐activated CSC signaling mediates the intestinal carcinogenesis following Smad4 loss and p53 loss.

### 3.6 Bcl‐X_L_ mediates Wnt/β‐catenin‐activated CSC signaling

**FIGURE 6:**
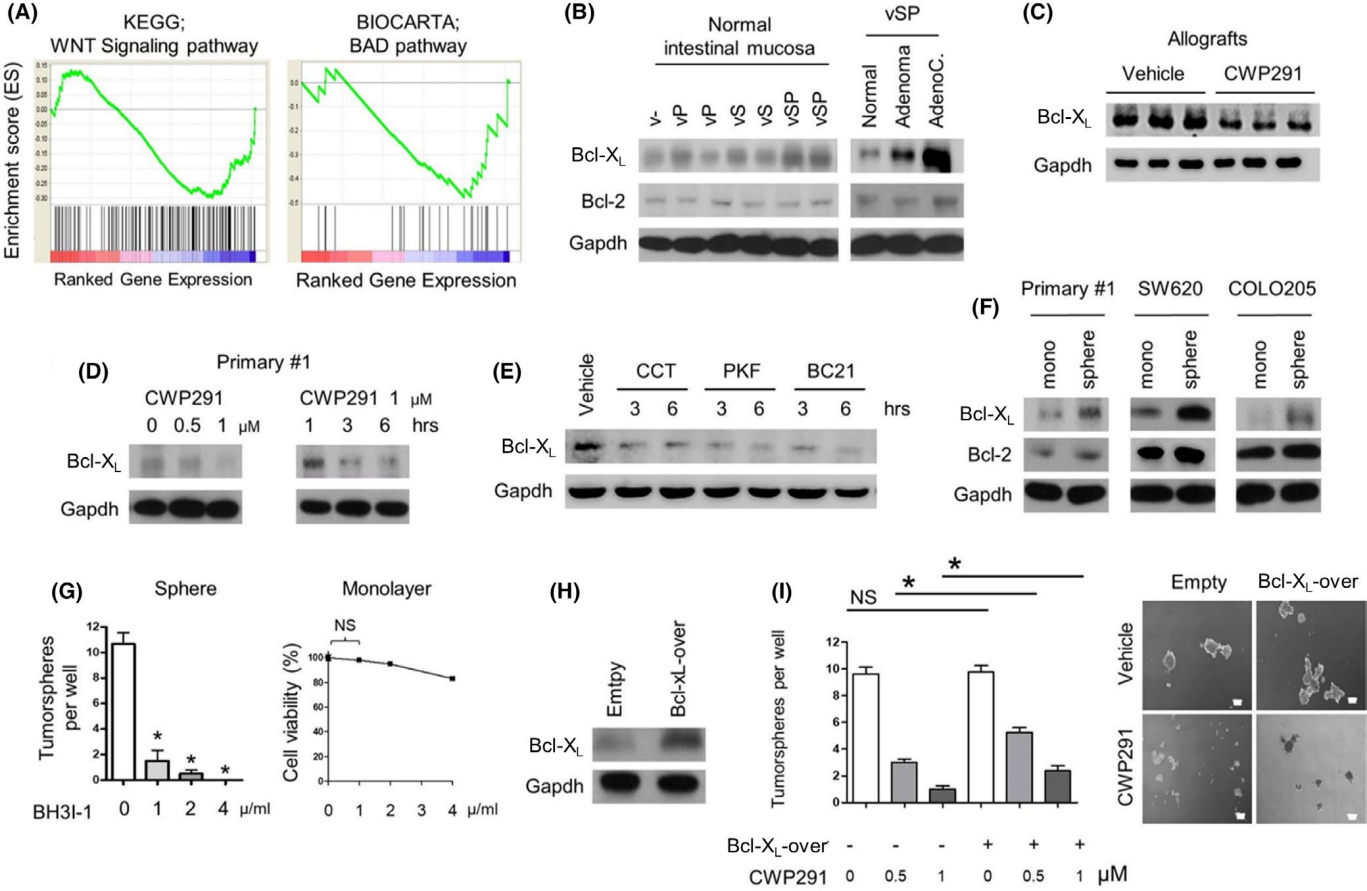
Downregulation of Bcl‐X_L_ after Wnt inhibition in intestinal cancer cells. (A) The downregulation of Wnt and BAD signaling pathway in CWP232291‐treated allografts (*n* = 2) and vehicle‐treated allografts (*n* = 2) using GSEA based on mouse gene expression microarray analysis. Either 150 mg/kg of CWP232291 or vehicle was administered to SCID mice biweekly for 8 weeks after subcutaneous injection of 1 × 10^6^ primary #1 cells. (B) Western blot analysis for Bcl‐X_L_ and Bcl‐2 in normal intestinal mucosa from each genotype and *Villin*‐*Cre*; *Smad4^F^* ^/^ *^F^*; *Trp53^F^* ^/^ *^F^* primary intestinal tumors. *vSP*, *Villin*‐*Cre*; *Smad4^F^* ^/^ *^F^*; *Trp53^F^* ^/^ *^F^*. vS, *Villin*‐*Cre*; *Smad4^F^* ^/^ *^F^*. *vP*, *Villin*‐*Cre*; *Trp53^F^* ^/^ *^F^*. AdenoC, adenocarcinoma. (C) Western blot analysis for Bcl‐X_L_ expression in CWP232291‐treated allografts (*n* = 3) and vehicle‐treated allografts (*n* = 3). (D) Reduced Bcl‐X_L_ expression in CWP232291‐treated primary #1 cells dose‐ and time‐dependently based on western blot analysis. (E) Reduced Bcl‐X_L_ expression in primary #1 cells after treatment with Wnt small‐molecule inhibitors based on western blot analysis. CCT, 20 µM CCT031374. PKF, 2 µM PKF 118–310. BC21, 10 µM BC21. (F) Western blot analysis for Bcl‐X_L_ and Bcl‐2 in monolayer and sphere cultures of primary #1, SW620, and COLO205 cells. (G) Sphere‐forming assay showing reduced tumorsphere formation after treatment of primary #1 cells with BH3I‐1, a Bcl‐X_L_ antagonist at a dose‐dependent manner. Right, the growth inhibitory effect of BH3I‐1 on monolayer culture of primary #1 cells was measured by MTT assay. (H) Bcl‐X_L_ overexpression in primary #1 cells as confirmed by western blot analysis. (I) Sphere‐forming assay in Bcl‐X_L_‐overexpressing primary #1 cells after CWP232291 treatment. Representative images for sphere‐forming assay were shown in the right. GSEA, gene set enrichment analysis; NS, No significance. *p < 0.05

We then further evaluated CSC signaling pathway components targeted by CWP232291 in *Villin*‐*Cre*; *Smad4^F^* ^/^ *^F^*; *Trp53^F^* ^/^ *^F^* primary cultured cells. According to GSEA analysis of DNA microarray data between CWP232291‐treated allografts (*n* = 2) and vehicle‐treated allografts (*n* = 2), the BCL2‐associated agonist of cell death (BAD) signaling pathway was enriched in differentially expressed genes (Figure 6A). Of several BAD signaling pathway components differentially expressed, Bcl‐X_L_ was given our primary focus because its mRNA expression level was higher in the intestinal mucosa deficient in both Smad4 and p53 than those deficient in either of the two genes. Bcl‐X_L_ was also overexpressed as cancer progresses in the background of Smad4 loss and p53 loss (Figure 6B).

According to the western blot analysis, Bcl‐X_L_ protein expression was reduced in allograft and primary cultured cells after CWP232291 treatment (Figure 6C,D). Bcl‐X_L_ was similarly downregulated in response to other Wnt inhibitors (Figure 6E; Figure S3A). SW620 and COLO205 tumorspheres overexpress Bcl‐X_L_ compared with monolayer culture (Figure 6F).

Importantly, treatment with BH3I‐1, a Bcl‐X_L_ antagonist, markedly suppressed the tumorsphere formation of primary #1 cells without affecting monolayer growth (Figure 6G). Reduced tumorsphere formation in CWP232291‐treated primary #1 cells was modestly rescued by Bcl‐X_L_ expression (Figure 6H,I). These results suggest that Bcl‐X_L_, at least partially, mediates the Wnt/β‐catenin‐activated CSC signaling in mouse intestinal adenocarcinomas deficient in Smad4 and p53.

## 4 DISCUSSION

*SMAD4* and *TP53* mutations are most common mutations in human CRC. Herein, we are the first to establish spontaneous colon cancer model mice with a *Villin*‐*Cre*; *Smad4^F^* ^/^ *^F^*; *Trp53^F^* ^/^ *^F^* background, confirming the outstanding occurrence of multiple spontaneous intestinal adenocarcinomas. We have shown that transcriptional activation of *Cdkn1a* (p21) and suppression of Wnt/β‐catenin pathway mediate the synergistic action in colorectal tumor suppression between Smad4 and p53. Our data are consistent with previous reports that Wnt pathway is activated by Smad4 loss^6^, ^28^, ^29^ and p53 loss.^30^, ^31^ Smad4 signaling reduces β‐catenin expression through miR‐139 in fibroblast.^6^ In neural crest cells, Smad4 loss downregulates Wnt pathway inhibitors Dkk1 and Sfrp1 and activates canonical WNT/β‐catenin signaling.^29^ In triple‐negative breast cancer, MET signaling plays a pivotal role in p53 loss‐induced Wnt activation.^30^ Expression of a set of canonical Wnt genes and Snail is reduced by p53 in CRC.^31^ It remains to be elucidated whether and how these potential mediators activate the complex Wnt/β‐catenin signaling network in our mouse model, which is one of limitations of this study. Also, screening microarray experiments were conducted on relatively small number of autochthonous intestinal cancers.

**FIGURE 7:**
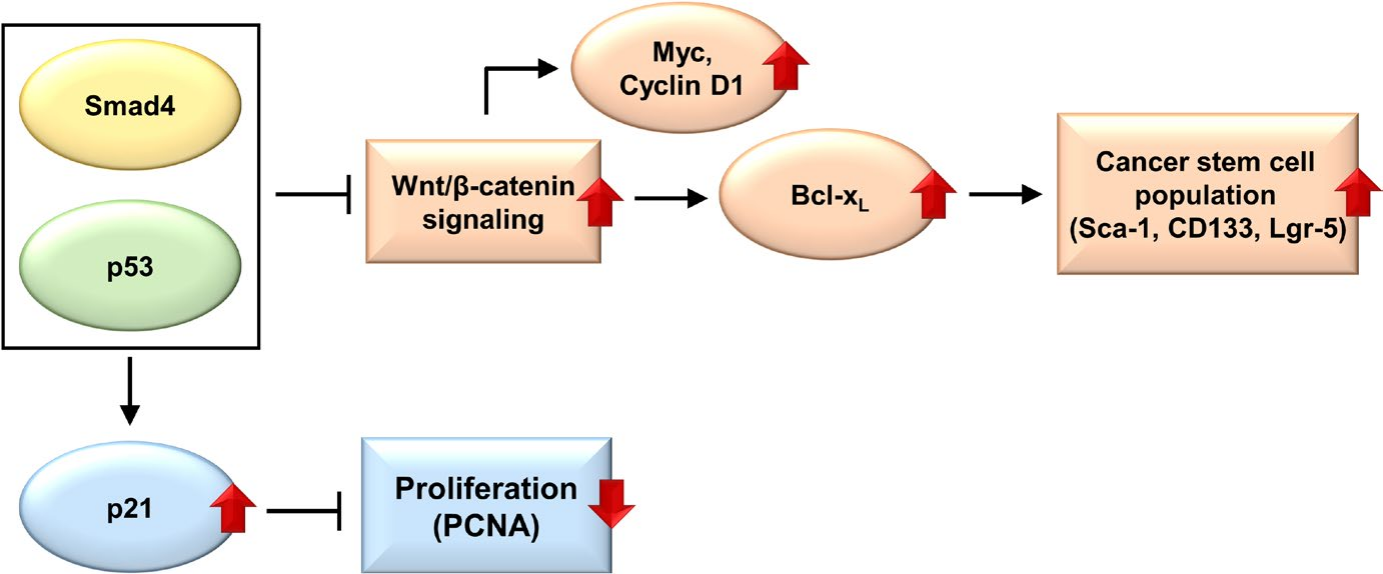
Downstream mediators of Smad4 loss and p53 loss in intestinal epithelium

Since both *SMAD4* and *TP53* mutations are inactivating mutations, they are not regarded as clinically actionable. Our mouse study, however, unequivocally demonstrated that a Wnt inhibitor reduces the incidence and invasiveness of autochthonous intestinal adenocarcinomas. Thus, this study provides proof‐of‐concept data that Wnt/β‐catenin inhibitors may suppress gastrointestinal cancers with *SMAD4* and *TP53* mutations that activate Wnt/β‐catenin pathway. In addition, we showed that β‐catenin‐induced Bcl‐X_L_ mediates CSC phenotypes in the gastrointestinal epithelium deficient in Smad4 and p53.^32^ Thus, our study validates and further elucidates the molecular link between Smad4 loss and p53 loss, Wnt/β‐catenin activation, and intestinal carcinogenesis, providing novel, clinically relevant insights into the *SMAD4* and *TP53* mutations in CRC. Thus, we conclude that Smad4 loss and p53 loss are synergistic in autochthonous intestinal carcinogenesis, through p21 inhibition and Wnt activation (Figure 7).

## ETHICS STATEMENT

All primary cell lines were obtained from autochthonous mouse tumor. All mouse experiments were performed in National Cancer Center in Korea and complied with animal ethics of IACUC (IACUC No. NCC‐21‐644).

## CONFLICT OF INTEREST

The authors have declared that no conflict of interest exists.

## AUTHOR CONTRIBUTIONS

Hark Kyun Kim and Jun Won Park devised the project, the main conceptual ideas, and proof outline. Jun Won Park, Min‐Jung Seo, and Kye Soo Cho processed the experimental data. Myeong‐Cherl Kook performed pathological analysis. Kye Soo Cho and Jong Min Jeong worked out laboratory animal management. Soo Young Cho performed statistical analysis. Soo Young Cho, Jae Hee Cheon, and Min‐Jung Seo wrote the article. Hark Kyun Kim, Min‐Jung Seo, and Seul‐Gi Roh performed revision response of the article.

## Supporting information

Supplementary Material

## Data Availability

The authors were unable to find a valid data repository for the data used in this study. These data are available from Hark Kyun Kim at National Cancer Center in Korea.
