# Supplementary material for: Smad4 and p53 synergize in suppressing autochthonous intestinal cancer
Source: Cancer Med. 2022 Mar 11;11(9):1925–36. doi: 10.1002/cam4.4533 (PMC9089223; doi:10.1002/cam4.4533)
Supplement: Supplementary file 1 — Supplementary Material [file CAM4-11-1925-s001.pdf]

Supplementary materials and methods

MICE

Mouse studies were conducted with the approval of the Animal Care and Use Committees of National Cancer Center of Korea. Villin-Cre (*B6.Cg-Tg(Vil-Cre)20Sy*) and *Trp53*$^{F/F}$ (*FVB.129-Trp53*^{tm1Brn}) mice were provided by the Mouse Models of Human Cancers Consortium. Conditional Smad4 knockout mice (*Smad4*$^{F/F}$) were previously described[1]. Compound conditional knockouts of Smad4 and p53 were bred with *Villin-Cre* mice to perform targeted deletion for these genes in intestinal epithelium. Offspring mice were genotyped using polymerase chain reaction (PCR) assays for tail DNA as described previously[1]. Mice positive for Villin-Cre genes were monitored until they became moribund or showed signs of distress, at which time necropsies were performed. Scheduled sacrifice was also conducted at 10, 15, and 20 weeks of age to evaluate the preneoplastic changes of intestinal mucosa. Intestines were fully examined according to the necropsy protocol described in our previous study[1]. Mesenteric lymph nodes, liver, and lung were harvested at necropsy to assess for metastases. Severe combined immunodeficiency (SCID) mice were purchased from Orient Bio (Seoungnam, Korea). Mouse studies were conducted with the approval of the Animal Care and Use Committees of National Cancer Center of Korea.

The intestine and excised tumors were fixed in neutral buffered 10% formalin, processed by standard methods and embedded in paraffin. H&E slides were interpreted by two pathologists independently according to the criteria (Fig. S2). All of H&E slides were scanned using Aperio ScanScope AT (Aperio Technologies, Inc., Vista, CA) to measure the thicknesses of intestinal epithelium.

For limiting dilution assays, mouse primary cancer cells (10^5, 10^4, and 10^3 cells) were suspended in 50 μl of RPMI and 50 μl of Matrigel (356234, BD Biosciences, San Jose) and were injected into the flank subcutaneous tissue of syngeneic (*Villin-Cre*-negative) mice. After 8 weeks of intraperitoneal CWP232291 (150mg/kg, twice per week) (JW Pharmaceutical, Seoul, Korea) or PBS treatment, the mice were euthanized and tumor formation was examined. The frequency of tumor-initiating cells (TICs) was calculated and P-value was evaluated using ELDA webtool (http://bioinf.wehi.edu.au/software/elda).

For ionizing radiation (IR) experiments, irradiation was performed using a 6 MV X-ray linear accelerator (Varain 600 CD, Palo Alto, CA USA). A single dose of 10 Gray (Gy) was given at a dose rate of 3 Gy/min on a rotating platform. After irradiation, the animals were taken back to the animal facility and were routinely cared for 24 h.

Cells

Mouse primary intestinal cancer cell lines used in this study were established from *Villin-Cre;Smad4$^{F/F}$;Trp53$^{F/F}$* mice. These cell lines were cultured at 37℃ in a 5% CO_2 humidified incubator in RPMI-1640 medium (Gibco, Grand Island, NY) containing 10% FBS (Gibco). The following reagents were used in cell experiments; BC21 (219334, Millipore, Billerica, MA), CCT031374 (#4675, R&D systems, Minnesota, USA), PKF 118-310 (219331; Calbiochem, San Diego, CA), BH3I-1 (sc221352, Santa Cruz Biotechnology Inc., Santa Cruz, CA), Etoposide (Sigma ,St.Luis, USA), and recombinant mouse Wnt3a (1324-WN, R&D Systems).

For tumorsphere formation, single cells were plated at 2,500 cell/ml in serum-free DMEM/F12 (Life Technologies, Gaithersburg, CA, USA), supplemented with 10

ng/ml fibroblast growth factor (R&D Systems), 20 ng/ml epidermal growth factor (R&D Systems), on UltraLow Attachment 24 well plates (Corning, Lowell, MA) for 7 days. Secondary tumorspheres were plated at 1,000 cells per well. Dishes were cultivated for 7 days to enumeration of spheres. Individual spheres ≥ 100 μm from each replicate well were counted using Axiovert 200M (Carl Zeiss, Oberkochen, Germany).

To measure Wnt activity, luciferase assays were carried out using the dual luciferase reporter assay system (Promega, Madison, WI). Wnt/β-catenin activity was evaluated by using Cignal TCF/LEF1 reporter plasmid (CCS-018L, SA Biosciences, Frederick, MD) according to the manufacture's instruction. For stable expression of Smad4, p53, and Bcl-X_L in primary cultured cells, we used lentiviral transduction system. *Trp53* and *Bcl2l1* genes were cloned into a modified lentiviral vector, named pCDH-CAG-MCS-EF1-Puro, from an original vector, pCDH-CMV-MCS-EF1-Puro (System Biosciences, CA, USA) as described in our previous report[2]. Smad4 gene was cloned into a vector, named pCDH-CAG-MCS-EF1-neo in which a puromycin selection marker was replaced with a neomycin selection marker. The gene cassettes of *Smad4*, *Trp53*, and *Bcl2l1* were amplified from cDNA of the mouse cells using PCR. All cloned genes were validated by using Sanger sequencing. Lentivirus was produced by co-transfecting lentiviral vectors and pMD2.G and psPAX2 constructs (Addgene, Watertown, MA) into 293T cells using lipofectamine 2000 (Invitrogen). Viral supernatants were harvested 48 hours after transfection, filtered through a 0.45 μm filter, and used to infect primary cultured cancer cells with 10 μg/mL polybrene. Cells were treated with 2 μg/mL of puromycin or 250 ug/ml of G418 at 48 hours after viral transduction and were selected for 3 days.

Supplementary Figure 1.

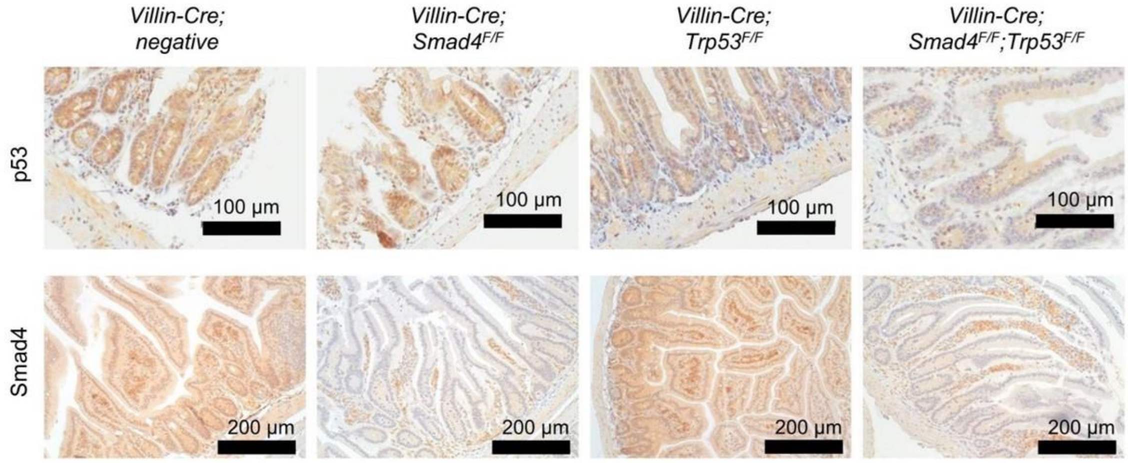

Fig S1. Immunohistochemical analysis of p53 and Smad4 on intestinal epithelium of each genotype. *Villin-Cre;Smad4*$^{F/F}$*;Trp53*$^{F/F}$ mice shows no immunoreactivity for p53 and Smad4 in intestinal epithelium.

Supplementary Figure 2.

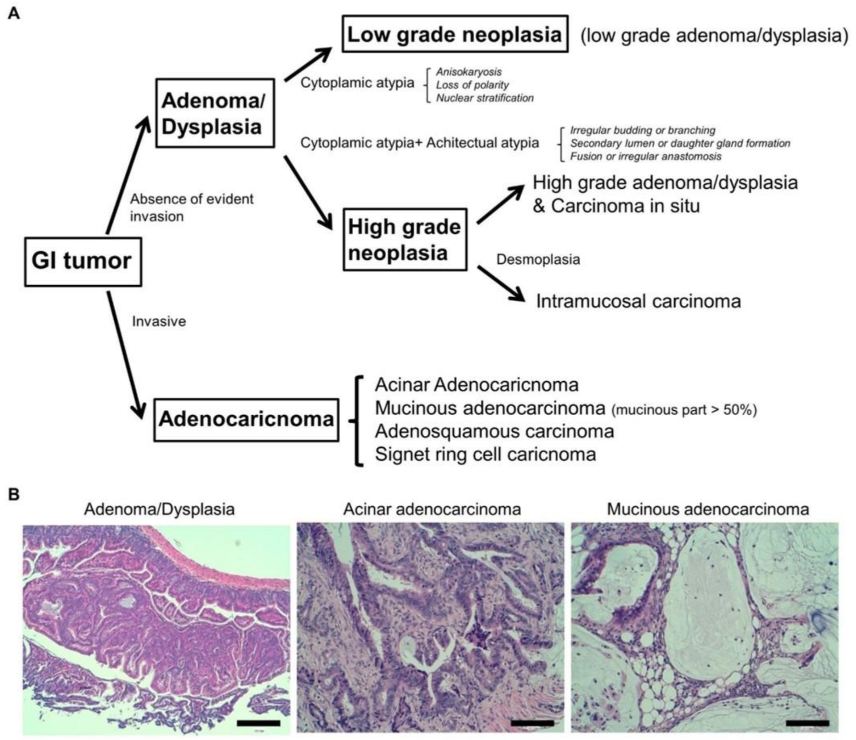

Fig S2. (A) Histological grading criteria for mouse intestinal tumors. (B) Representative H&E images for neoplastic intestinal lesions of *Villin-Cre;Smad4*$^{F/F}$*;Trp53*$^{F/F}$ mice. Bar = 100 μm

Supplementary Figure 3.

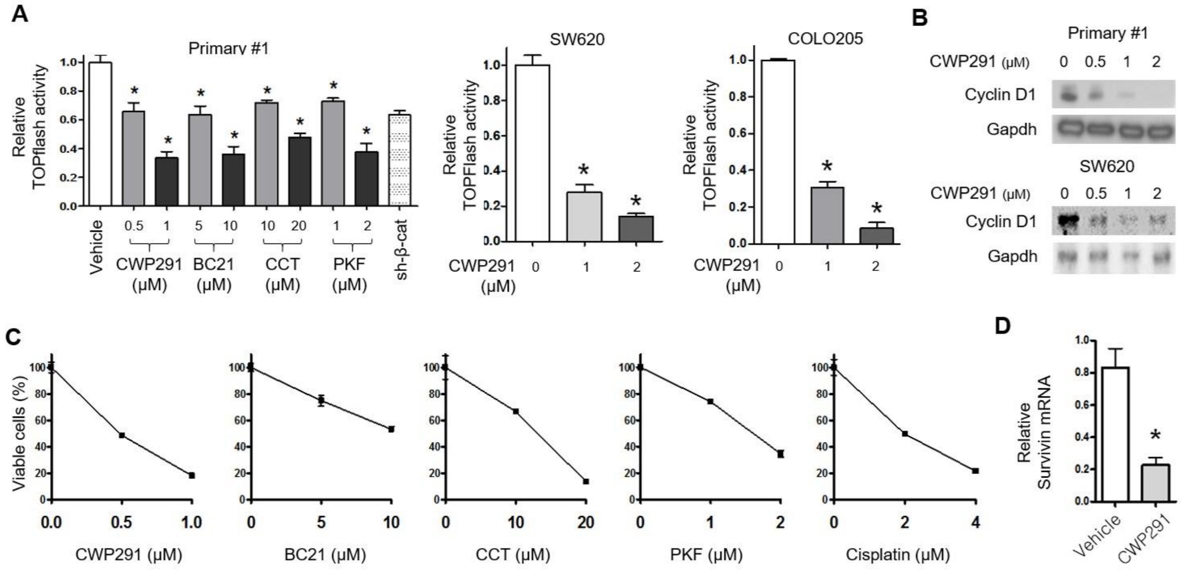

Fig S3. Effect of WNT inhibitors on the cell growth and CSC surivival in double knockout cells. CWP291, CWP232291. CCT, CCT031374. PKF, PKF 118-310. (A) Attenuated TOPflash reporter activities after 12 hours of treatment with WNT inhibitors at the indicated doses in primary #1 cell line, human colorectal

cancer cell lines SW620 and COLO205. (B) The inhibitory effect of CWP232291 on the expression of cyclin D1 in primary #1 and SW620 based on western blot analysis. (C) MTT assay to measure growth-inhibitory effects of WNT inhibitors in primary #1 cells. Cells were treated with WNT inhibitors for 48 hours at the indicated doses. 24 hours before the treatment, 0.25 x 105 cells were seeded on 6 well cell culture plates. (D) Quantitative real-time RT PCR analysis for mRNA expression of survivin after 24 hours of in-vivo treatment with 100 mg/kg CWP232291 via intraperitoneal injection. (*$P<0.05$, **$P<0.01$)

Supplementary Table 1. DAVID pathway analysis on the genes up-regulated by > 2-fold in intestinal adenocarcinomas arising from Villin-Cre;Smad4F/F;p53F/F mice (n=2) compared with normal intestinal mucosa from Villin-Cre-negative mice (n=2).

KEGG_PATHWAY	P Value	Genes
mmu04510:Focal adhesion	0.000	PDGFB, PGF, PIP5K1C, CHAD, ILK, PDGFC, PDGFD, AKT3, SHC4, PIK3CG, EGFR, PARVG, ACTN1, FLNC, FLNA, PRKCB, COL1A2, PDGFRA, PDGFRB, RELN, LAMC2, LAMC1, COL1A1, PARVB, IBSP, CAV2, CAV1, TNC, COL3A1, ITGB5, ITGB3, MYL9, IGF1R, LAMB2, RAC2, ITGB8, RAC3, ITGAV, COL6A1, PIK3R5, THBS1, FIGF, THBS2, FN1, THBS4, SPP1, COL4A2, COL4A1, FLT1, FLT4, ITGA1, IGF1, ITGA4, HGF, CAPN2, COL5A2, VAV1, KDR, LAMA2, LAMA1, ITGA9, VWF, LAMA4, LAMA3,

		ITGA5, LAMA5, FYN, ITGA8
mmu04060:Cytokine-cytokine receptor interaction	0.000	PDGFB, IL6ST, OSMR, LEPR, IL18, TGFB3, CXCR2, TNFSF18, TGFB1, TGFB2, TNFRSF11B, CXCR4, CSF2RB, CSF3R, IL1B, PDGFC, PDGFD, CSF2RA, IL1A, GHR, EGFR, IL18RAP, LIFR, CCL21A, OSM, INHBB, TNFRSF9, IFNAR2, INHBA, PPBP, CCR5, CCR2, PDGFRA, PDGFRB, IL1R2, CCL3, IL1R1, TNF, CCL2, CXCL5, TNFRSF12A, CSF2RB2, CRLF2, CCR1, CSF1, IL4RA, CXCL2, CCL9, BMPR2, CCL8, PF4, IL7R, CCL7, LIF, TNFRSF1B, IL10RA, IL2RG, FIGF, CSF1R, IL18R1, IL6, FLT1, TGFBR1, FLT4, TGFBR2, HGF, TNFSF9, CCL17, TNFSF8, IL6RA, KDR, CCL11, CXCL14, CXCL13, CXCL16
mmu04610:Complement and coagulation cascades	0.000	C7, C3AR1, A2M, MASP1, C3, F13A1, BDKRB1, C1S, C1QC, C1RA, C1RB, SERPINA1B, SERPINE1, CFH, C2, PLAT, KNG1, KNG2, F10, C5AR1, C4B, SERPING1, SERPINA1E, F7, PLAUR, C1QA, C1QB, VWF, GM5077, C4BP, THBD, CD59B, CD59A, F3, TFPI, PROS1, PLAU, F2R
mmu04512:ECM-receptor interaction	0.000	IBSP, TNC, NPNT, COL3A1, ITGB5, ITGB3, SDC3, CHAD, CD47, LAMB2, ITGB8, ITGAV, COL6A1, THBS1, THBS2, SPP1, THBS4, FN1, COL4A2, COL4A1, ITGA1, ITGA4, COL5A2, LAMA2, VWF, ITGA9, LAMA1, LAMA4, CD36, LAMA3, ITGA5, LAMA5, ITGA8, COL1A2, RELN, LAMC2, COL1A1, LAMC1
mmu04640:Hematopoietic cell lineage	0.000	IL1R2, IL1R1, TNF, CSF1, IL4RA, ANPEP, ITGB3, IL7R, ITGAM, CD22, CSF3R, IL1B, IL1A, CSF2RA, CSF1R, IL6, ITGA1, ITGA4, FCGR1, IL6RA, CD37, CD36, CD59B, CD34, ITGA5, CD59A, CD33, H2-AA, CD14
mmu04670:Leukocyte transendothelial migration	0.000	CLDN8, CLDN4, MMP9, SIPA1, ITGB2, MMP2, ITGAM, CDH5, MYL9, VCAM1, RAC2, CXCR4, PIK3R5, ESAM, MSN, PIK3CG, ICAM1, NCF2, NCF1, NCF4, ACTN1, MAPK11, ITGA4, VAV1, THY1, PRKCB, CYBA, CYBB, RASSF5, PECAM1, CLDN1, JAM2, JAM3
mmu04810:Regulation of actin	0.000	FGFR1, ENAH, PDGFB, MRAS, ITGB5, PIP5K1C, ABI2, FGF10, ITGB2, RDX, BDKRB1, ITGB3, ITGAM, MYL9,

cytoskeleton		PFN2, RAC2, ITGAX, ITGB8, RAC3, ITGAV, RRAS, PDGFC, PIK3R5, MSN, PDGFD, FGF2, FN1, PIK3CG, EGFR, ARHGEF6, ITGA1, ACTN1, NCKAP1L, ITGA4, ARHGEF12, VAV1, ITGA9, CHRM2, ITGA5, ITGA8, PDGFRA, PDGFRB, PIP4K2A, CD14, MYH10, PIP4K2B, F2R
mmu04514:Cell adhesion molecules (CAMs)	0.000	CLDN8, CLDN4, ITGB2, CDH2, CDH3, ITGAM, CDH5, SDC3, ALCAM, VCAM1, ITGB8, ITGAV, CD22, ESAM, SELPLG, PTPRC, ICAM1, SELP, PTPRM, SELL, NFASC, NLGN2, ITGA4, NCAM1, SIGLEC1, ITGA9, CD86, CD80, CD34, ITGA8, CD274, PECAM1, CLDN1, H2-AA, VCAN, JAM2, JAM3
mmu05410:Hypertrophic cardiomyopathy (HCM)	0.000	CACNA2D1, IL6, TNF, ITGA1, TGFB3, IGF1, ITGB5, ITGA4, ITGB3, TPM2, TGFB1, TPM4, TGFB2, LAMA2, ITGA9, ACE, DES, ITGA5, ITGB8, ITGA8, ITGAV, CACNA1C, CACNA1D, SGCB
mmu05414:Dilated cardiomyopathy	0.000	ADCY4, TNF, ADCY7, TGFB3, ITGB5, ITGB3, TPM2, TGFB1, TPM4, TGFB2, DES, ITGB8, ITGAV, PRKACB, CACNA2D1, ITGA1, IGF1, ITGA4, LAMA2, ITGA9, ITGA5, ITGA8, CACNA1C, CACNA1D, SGCB
mmu04062:Chemokine signaling pathway	0.000	ADCY4, CCL3, CCL2, ADCY7, CXCL5, CCR1, CXCL2, CCL9, CCL8, CXCR2, PF4, CCL7, DOCK2, RAC2, CXCR4, GNG2, PIK3R5, PRKACB, AKT3, SHC4, PIK3CG, LYN, NCF1, CCL21A, VAV1, PRKCB, CCL17, CCL11, GNGT2, ARRB2, CCR5, PPBP, CXCL14, CXCL13, CXCL16, CCR2, GNB4, GRK5
mmu05200:Pathways in cancer	0.000	PTGS2, PDGFB, PGF, MMP9, MITF, TGFB3, FGF10, MMP2, GLI3, TGFB1, TGFB2, CSF3R, RARB, FGF2, MYC, AKT3, CSF2RA, PIK3CG, EGFR, AR, RUNX1T1, PRKCB, HIF1A, PDGFRA, PDGFRB, LAMC2, LAMC1, FGFR1, SFPI1, ZBTB16, IGF1R, LAMB2, RAC2, RAC3, ITGAV, PIK3R5, RUNX1, FIGF, CSF1R, FN1, BMP4, IL6, COL4A2, COL4A1, TGFBR1, TGFBR2, FZD1, IGF1, HGF, FZD7, LAMA2, LAMA1, CBLB, LAMA4, RASSF5, LAMA3, LAMA5
mmu04010:MAPK signaling pathway	0.000	MEF2C, PDGFB, TGFB3, FGF10, TGFB1, TGFB2, MAP3K6, MAP3K8, RRAS, IL1B, PRKACB, MYC, FGF2,

		AKT3, IL1A, EGFR, FLNC, FLNA, PRKCB, MAP4K4, ARRB2, GADD45G, PDGFRA, PLA2G2A, PDGFRB, HSPB1, FGFR1, IL1R2, IL1R1, TNF, TM4SF19, MRAS, RAC2, RASGRP3, RAC3, CACNA2D1, TGFBR1, TGFBR2, NR4A1, MAPK11, DUSP4, PLA2G4A, DUSP1, RPS6KA2, CACNA1C, CACNA1D, DUSP7, CD14, DUSP6
mmu05020:Prion diseases	0.000	EGR1, C7, IL6, C1QC, NCAM1, C1QA, C1QB, FYN, CASP12, IL1B, LAMC1, PRKACB, IL1A
mmu04270:Vascular smooth muscle contraction	0.000	ADCY4, ADCY7, CALD1, PRKG1, KCNMB1, MYL9, EDNRA, KCNU1, ACTG2, GUCY1A2, GUCY1A3, CALCRL, PRKACB, RAMP2, ACTA2, PRKCH, NPR2, ARHGEF12, PRKCB, PLA2G4A, AVPR1A, MYH11, PLA2G2A, GUCY1B3, CACNA1C, CACNA1D
mmu04666:Fc gamma R-mediated phagocytosis	0.001	PIK3CG, PTPRC, DNM3, PLD2, LYN, MARCKSL1, NCF1, SPHK1, PIP5K1C, VAV1, FCGR1, PRKCB, DOCK2, PLA2G4A, RAC2, FCGR2B, PIK3R5, MARCKS, INPP5D, PPAP2B, AKT3, PIP4K2B
mmu04662:B cell receptor signaling pathway	0.001	PIK3CG, TM4SF19, LYN, IFITM1, CD72, VAV1, BTK, PRKCB, RASGRP3, FCGR2B, DAPP1, RAC2, RAC3, LILRB3, CD22, PIK3AP1, PIK3R5, INPP5D, AKT3
mmu00532:Chondroitin sulfate biosynthesis	0.001	CSGALNACT1, UST, CHST12, CHST11, CSGALNACT2, CHST14, CHSY1, CHST15, DSE
mmu05412:Arrhythmogenic right ventricular cardiomyopathy (ARVC)	0.001	CACNA2D1, ITGA1, GJA1, ACTN1, ITGB5, CDH2, ITGB3, ITGA4, LAMA2, ITGA9, DES, ITGB8, ITGA5, ITGAV, ITGA8, CACNA1C, CACNA1D, SGCB
mmu04630:Jak-STAT signaling pathway	0.002	OSMR, CSF2RB2, IL6ST, LEPR, CRLF2, IL4RA, IL7R, SPRY4, LIF, SPRY1, IL10RA, CSF3R, CSF2RB, IL2RG, PIK3R5, SPRED1, MYC, AKT3, CSF2RA, GHR, PIK3CG, IL6, SOCS3, PIM1, LIFR, IL6RA, OSM, IFNAR2, CBLB
mmu04540:Gap junction	0.002	EGFR, ADCY4, TUBB2B, PDGFB, ADCY7, GJA1, PRKG1, PRKCB, GUCY1A2, PDGFRA, GUCY1A3, PDGFRB, TUBB6, GUCY1B3, PDGFC, PDGFD, PRKACB, TUBA1A, HTR2B
mmu04350:TGF-beta signaling	0.003	BMP4, LTBP1, TNF, TGFBR1, FST, TGFBR2, BMPR2, TGFB3, DCN, TGFB1, TGFB2, INHBB, INHBA, ID2, THBS1,

pathway		MYC, THBS2, BMP8B, THBS4
mmu05218:Melanoma	0.005	EGFR, PIK3CG, FGFR1, PDGFB, MITF, IGF1, FGF10, HGF, IGF1R, PDGFRA, PDGFRB, PIK3R5, PDGFC, PDGFD, FGF2, AKT3
mmu05222:Small cell lung cancer	0.005	PIK3CG, COL4A2, COL4A1, PTGS2, LAMA2, LAMA1, LAMA4, LAMB2, LAMA3, LAMA5, ITGAV, PIK3R5, LAMC2, LAMC1, RARB, MYC, AKT3, FN1
mmu04664:Fc epsilon RI signaling pathway	0.009	PIK3CG, TNF, LYN, MAPK11, VAV1, PRKCB, BTK, PLA2G4A, RAC2, RAC3, FYN, PLA2G2A, FCER1G, PIK3R5, INPP5D, AKT3, LCP2
mmu04142:Lysosome	0.009	TCIRG1, HYAL1, PLA2G15, GM2A, GUSB, LGMN, HEXB, CTSS, GNS, SLC11A1, CTSL, CTSK, CD68, LAPTM5, AP1S2, CTSE, GALC, CTSD, CTSC, MAN2B1, ATP6V0D2, CTSH
mmu00603:Glycosphingolipid biosynthesis	0.010	ST3GAL2, A4GALT, FUT9, GBGT1, B3GALNT1, HEXB
mmu04620:Toll-like receptor signaling pathway	0.012	PIK3CG, CCL3, IL6, TNF, LY96, MAPK11, TLR7, TLR8, IFNAR2, CD86, IRF5, CD80, MAP3K8, IL1B, PIK3R5, LBP, CD14, AKT3, SPP1
mmu05210:Colorectal cancer	0.014	EGFR, PIK3CG, TGFBR1, TGFBR2, FZD1, TGFB3, FZD7, TGFB1, TGFB2, IGF1R, RAC2, RAC3, PDGFRA, PDGFRB, PIK3R5, MYC, AKT3
mmu05322:Systemic lupus erythematosus	0.017	C7, TNF, C3, C4B, FCGR4, ACTN1, C1S, C1QC, FCGR1, FCGR3, C1QA, C1QB, C1RA, C1RB, GM5077, CD86, CD80, FCGR2B, H2-AA, C2
mmu04650:Natural killer cell mediated cytotoxicity	0.024	PIK3CG, ICAM1, TNF, TM4SF19, FCGR4, ITGB2, VAV1, FCGR3, PRKCB, CD48, IFNAR2, RAET1D, RAC2, RAC3, FYN, FCER1G, PIK3R5, SH3BP2, TYROBP, SHC4, LCP2
mmu05212:Pancreatic cancer	0.031	PIK3CG, EGFR, PGF, ARHGEF6, TGFBR1, TGFBR2, TGFB3, TGFB1, TGFB2, RAC2, RAC3, PIK3R5, FIGF, AKT3
mmu04020:Calcium signaling pathway	0.034	GNA14, ADCY4, TM4SF19, CYSLTR1, ADCY7, TACR1, BDKRB1, ITPKB, EDNRA, EDNRB, ATP2B4, PDE1A, PRKACB, EGFR, SLC25A4, BST1, SPHK1, PRKCB, ADRB2, P2RX7, CHRM2, AVPR1A, PDGFRA, PDGFRB,

		CACNA1C, HTR2B, CACNA1D, PTAFR, F2R
mmu05215:Prostate cancer	0.041	PIK3CG, EGFR, FGFR1, AR, PDGFB, IGF1, CREB5, IGF1R, CREB3L2, PDGFRA, PDGFRB, PIK3R5, PDGFC, PDGFD, SRD5A2, AKT3
mmu04370:VEGF signaling pathway	0.046	PIK3CG, TM4SF19, PTGS2, SPHK1, MAPK11, PRKCB, KDR, PLA2G4A, RAC2, RAC3, PLA2G2A, HSPB1, PIK3R5, AKT3
mmu04621:NOD-like receptor signaling pathway	0.050	CCL11, IL6, TNF, CCL2, IL18, CXCL2, PSTPIP1, IL1B, CCL8, MAPK11, NLRP3, CCL7
mmu04210:Apoptosis	0.060	PIK3CG, IL1R1, TNF, TM4SF19, CSF2RB2, CAPN2, PRKAR2B, IRAK3, CASP12, CSF2RB, IL1B, PIK3R5, PRKACB, AKT3, IL1A
mmu04360:Axon guidance	0.078	PLXNC1, NRP1, PLXNA4, TM4SF19, FES, ARHGEF12, SLIT2, EPHA3, SLIT3, RAC2, RAC3, CXCR4, FYN, SEMA7A, SEMA3D, SEMA4C, EFNA5, SEMA3A, SEMA4D, SRGAP2
mmu02010:ABC transporters	0.086	ABCC9, ABCA9, ABCB1B, ABCA8A, ABCD2, ABCC4, ABCA1, ABCA6, ABCG1
mmu04520:Adherens junction	0.088	EGFR, PTPRB, FGFR1, PTPRM, TGFBR1, TGFBR2, ACTN1, SNAI1, IGF1R, RAC2, FYN, RAC3, PTPN1
mmu04144:Endocytosis	0.094	PIP5K1C, CXCR2, IGF1R, DAB2, CXCR4, IL2RG, EHD2, EHD3, CSF1R, EGFR, FAM125B, DNM3, PLD2, FLT1, TGFBR1, TGFBR2, KDR, RAB11FIP5, ADRB2, RAB31, CBLB, ARRB2, CCR5, SH3KBP1, PDGFRA, GRK5, F2R, PIP4K2B
mmu04150:mTOR signaling pathway	0.099	PIK3CG, EIF4EBP1, HIF1A, PGF, RPS6KA2, IGF1, PIK3R5, FIGF, AKT3, DDIT4

Supplementary Table 2. Tumor-initiating ability according to Sca1 expression in primary mouse intestinal cancer cell lines

**No. of Injected cells**	**Primary #1**		**P Value**	**Primary #2**		**P value**
	**Neg.**	**Positive**		**Low**	**High**	
**1 X 10^5**	80% (4/5)	100% (5/5)	1.39e-05	100% (5/5)	100% (5/5)	0.019
**1 X 10^4**	60% (3/5)	100% (5/5)		80% (4/5)	100% (5/5)	
**1 X 10^3**	20% (2/5)	60% (3/5)		60% (3/5)	80% (4/5)	
**TIC * Frequency**	1/25889	1/1090		1/3596	1/622	

TIC frequency was calculated and p value was measured using ELDA webtool.

Abbreviation: TIC, tumor-initiating cell.

Supplementary Table 3. DAVID pathway analysis on the genes down-regulated by > 1.5-fold in

CWP291-treated allograft (n=2) compared with vehicle-treated allograft (n=2).

KEGG_PATHWAY	P-Value	Genes
Cytokine-cytokine receptor interaction	0.00000	CCL2, LEPR, CXCL9, TGFB3, IL15, CCL5, TNFSF18, TGFB2, CCL22, XCR1, IL2RB, IL2RA, TGFBR2, MET, LIFR, KDR, CCL17, CCL11, TNFRSF9, CCR7, TNFSF10, TNFSF13B, PRLR, CCR2, PDGFRA, NGFR, BMPR1B, XCL1
Graft-versus-host disease	0.00008	KLRA9, H2-M2, LOC641240, KLRA14, H2-AA, H2-T22, GZMB, H2-Q6, KLRD1, H2-DMB2
Cell adhesion molecules (CAMs)	0.00011	CLDN8, H2-M2, L1CAM, CDH2, H2-Q6, SDC2, H2-DMB2, SDC3, NCAM1, LOC641240, H2-T22, H2-AA, CD4, JAM3
Type I diabetes mellitus	0.00089	H2-M2, LOC641240, H2-AA, H2-T22, GZMB, H2-Q6, PTPRN, H2-DMB2
Antigen processing and presentation	0.00180	H2-M2, LOC641240, H2-AA, H2-T22, CD4, H2-Q6, KLRD1, H2-DMB2, KIR3DL1
Intestinal immune network for IgA production	0.00210	TNFSF13B, LOC641240, TGFB3, H2-AA, IL15, H2-DMB2, TGFB2
Viral myocarditis	0.00220	LAMA2, CAV1, H2-M2, LOC641240, H2-AA, H2-T22, H2-Q6, H2-DMB2, MYH10
Allograft rejection	0.00300	H2-M2, LOC641240, H2-AA, H2-T22, GZMB, H2-Q6, H2-DMB2
ECM-receptor interaction	0.00440	LAMA2, TNC, ITGA11, RELN, COL11A1, COL5A2, SDC2, SDC3
Chemokine signaling pathway	0.00550	CCL11, CCR7, CCL22, CCL2, CCR2, CXCL9, GRK5, XCL1, CCL5, XCR1, AKT3, CCL17
Autoimmune thyroid disease	0.00870	H2-M2, LOC641240, H2-AA, H2-T22, GZMB, H2-Q6, H2-DMB2
Focal adhesion	0.01000	LAMA2, CAV1, TLN2, TNC, MET, ITGA11, PDGFRA, RELN, COL11A1, COL5A2, AKT3, KDR

Endocytosis	0.01200	IL2RB, IL2RA, FGFR3, H2-M2, MET, TGFBR2, PDGFRA, H2-T22, GRK5, H2-Q6, F2R, KDR
Colorectal cancer	0.02000	MET, TGFBR2, TGFB3, FZD1, PDGFRA, AKT3, TGFB2
Natural killer cell mediated cytotoxicity	0.03200	CD48, TNFSF10, KLRA9, SH2D1B1, KLRA14, KLRK1, GZMB, NCR1, KLRD1
Jak-STAT signaling pathway	0.03500	STAT4, IL2RB, SPRY1, IL2RA, PRLR, LEPR, LIFR, IL15, AKT3
Complement and coagulation cascades	0.03800	FGG, FGB, C3, C4B, SERPING1, F2R
Axon guidance	0.04400	SEMA5A, EPHA7, PLXNB1, SEMA7A, MET, SEMA3D, L1CAM, SLIT3
Asthma	0.04800	CCL11, LOC641240, H2-AA, H2-DMB2

Supplementary References

1. Deng M, Hou SY, Tong BD, Yin JY, Xiong W. The Smad2/3/4 complex binds miR-139 promoter to modulate TGFbeta-induced proliferation and activation of human Tenon's capsule fibroblasts through the Wnt pathway. *J Cell Physiol.* 2019;234:13342-13352.

2. Park JW, Jang SH, Park DM, et al. Cooperativity of E-cadherin and Smad4 loss to promote diffuse-type gastric adenocarcinoma and metastasis. *Mol Cancer Res.* 2014;12:1088-1099.
